# A Randomized Controlled Trial Comparing Two Doses of Caffeine for Apnoea in Prematurity

**DOI:** 10.3390/ijerph18094509

**Published:** 2021-04-23

**Authors:** Anis Munirah Mohd Kori, Hans Van Rostenberghe, Nor Rosidah Ibrahim, Najib Majdi Yaacob, Ariffin Nasir

**Affiliations:** 1 Department of Paediatrics, Health Campus, School of Medical Sciences, Universiti Sains Malaysia, Kubang Kerian 16150, Kelantan, Malaysia; munirah_anis@student.usm.my (A.M.M.K.); hansvro@usm.my (H.V.R.); nrosidah@usm.my (N.R.I.); 2 Hospital USM, Universiti Sains Malaysia, Kubang Kerian 16150, Kelantan, Malaysia; 3 Department of Biostatistics and Research Methodology, Health Campus, School of Medical Sciences, Universiti Sains Malaysia, Kubang Kerian 16150, Kelantan, Malaysia; najibmy@usm.my

**Keywords:** apnoea, prematurity, caffeine, neonatal morbidities

## Abstract

Caffeine is the most commonly used methyl xanthine for the prevention of apnoea in prematurity, but the ideal dose was uncertain, until now. This study compared two doses of caffeine for the prevention of apnoea in prematurity. A clinical trial was conducted on 78 preterm infants ≤32 weeks in Neonatal Intensive Care Unit. They were randomly allocated to receive the intervention (loading 40 mg/kg/day and maintenance of 20 mg/kg/day) or the control (loading 20 mg/kg/day and maintenance of 10 mg/kg/day) dose of caffeine. The primary outcome of the study was the frequency and total days of apnoea per duration of treatment for both groups. The frequency of apnoea ranged from zero to fourteen in the intervention group and zero to twelve in the control group. There was no statistically significant difference between the groups, with a *p*-value of 0.839. The number of days of apnoea was also similar between both groups, with a *p*-value of 0.928. There was also no significant difference in adverse events between both regimens. This study did not support the use of higher doses of caffeine as a prevention for apnoea in prematurity.

## 1. Introduction

Apnoea of prematurity is a significant clinical problem reflecting the immaturity of respiratory control systems [1,2]. The incidence is widely variable depending on the gestational age. Uncontrolled episodes of apnoea maybe associated with prolonged ventilation, failed extubation, and prolonged hospital stay [3]. A Cochrane review showed that caffeine was superior to other methylxanthines for the prevention and treatment of apnoea in prematurity in view of its lower toxicity and wider therapeutic index [4].

The caffeine for apnoea in prematurity (CAP) trial has shown that the benefits of caffeine therapy outweigh any potential risk up to the corrected age of 18 to 24 months [5]. A subsequent follow-up of the CAP trial has shown that caffeine therapy reduces the severity of motor impairments at five years of corrected age [6]. Caffeine is now widely used as a standard treatment for apnoea in prematurity. Side effects of caffeine are relatively rare, but include tachycardia, jitteriness, feeding intolerance, increased oxygen consumption, and transient decrease in the rate of growth [2,7].

Despite the establishment of caffeine therapy in preterm infants, variable information exists on the optimal loading and maintenance dose in different neonatal departments. A commonly used dose for caffeine citrate is a 20 mg/kg loading followed by 5 to 10 mg/kg per day as maintenance [8]. The optimum caffeine dose in preterm infants has not been well-studied in terms of benefits and risks. There are two meta-analyses conducted comparing different dosages of caffeine. Even though a few trials have showed benefits of giving higher doses of caffeine without causing adverse side effects, the results of the meta-analyses were inconclusive [3,9,10]. The reason for conducting this study was to determine the efficacy and safety of a high versus a lower dose of caffeine for the prevention of apnoea in prematurity.

## 2. Materials and Methods

This study was a parallel, randomized controlled trial with a one-to-one allocation ratio. The trial has been registered with the ACTRN12619001708145 and no changes have been made to the method after trial commencement. This trial was approved by the Research and Ethics Committee School of Medical Sciences, Universiti Sains Malaysia (USM/JEPeM/18120788).

Infants admitted to the NICU of Hospital Universiti Sains Malaysia (USM) between June 2019 and August 2020 with a gestational age of ≤32 weeks were eligible to be included in the study. The exclusion criteria were Hydrops fetalis, a major congenital anomaly as defined by Garne et al. [11]. All infants meeting the inclusion and exclusion criteria were enrolled in this study. Hospital USM is a tertiary teaching hospital in the north-eastern part of Peninsular Malaysia.

Enrolled infants were randomly assigned to one of the two study groups. Infants in the intervention group received a high-dose regimen of oral caffeine citrate (loading dose of 40 mg/kg/day and maintenance dose of 20 mg/kg/day), whereas infants in the control group received a lower dose regimen of oral caffeine (loading dose of 20 mg/kg/day and maintenance dose of 10 mg/kg/day). Caffeine citrate was diluted for both groups into equal volume (loading 2 mL/kg and maintenance 1 mL/kg). Caffeine was continued until the age of 34 weeks. Based on the judgement of the treating neonatologist, the treatment could be prolonged or shortened.

The primary outcome of the study was the frequency and number of days with apnoeic episodes throughout the duration of caffeine therapy. Apnoea was defined as the cessation of breathing for more than 20 s and accompanied by hypoxia and/or bradycardia [1].

Secondary outcomes included extubation failure, duration of non-invasive ventilation (e.g., optiflow, Bipap, Duopap and Cpap), duration of oxygen therapy (nasal prong oxygen), chronic lung disease of a new-born defined, as the need for oxygen treatment at 36 weeks’ postmenstrual age [12], and the need for post-natal steroid therapy.

Other secondary outcomes included tachycardia (>170 beats/minute) [13], hypertension, time to reach full enteral feeding, urine output, weight gain, development of osteopenia of prematurity, length of hospital stay, neonatal mortality, necrotizing enterocolitis [14], intraventricular haemorrhage (IVH), periventricular leukomalacia (PVL), retinopathy of prematurity (ROP), and the number of infants for whom caffeine was withheld early because of suspected side effects.

Weaning from mechanical ventilation, non-invasive ventilation, and supplemental oxygen was based on the judgement of the treating neonatologist. Infant’s oxygen saturation, heart rate, and respiratory rate were continuously monitored, with the targeted oxygen saturation maintained between 91 and 95%. Caffeine was withheld by the treating neonatologist when significant side effects of caffeine were suspected. Full enteral feeding is defined as oral feeding of at least 100 mL/kg/day. Urine output was indirectly measured by weighing the diapers. The weight of the infant was recorded twice weekly. Osteopenia of prematurity was diagnosed in infants with ALP >500IU/L and serum phosphate level <1.8 mmol/L [15]. One outcome was added after data collection: urine electrolytes including urine calcium and phosphate, which were measured at day 7 of caffeine treatment in ten infants.

The sample size was estimated as follows. If the response within each subject group was normally distributed with a standard deviation of 2.75 and the true difference in the experimental and control means would be two apnoea episodes, we needed to study 78 subjects to achieve a power of 0.8 with alpha of 0.05 and 20% dropouts.

The random sequence was generated by a researcher not involved in the recruitment of patients and data collection using blocks of variable sizes known only to the randomizer. Concealment of allocation was ensured by the used of opaque, sealed and sequentially numbered envelopes. A designated pharmacist prepared the caffeine and was the only person not blinded to the allocation. Patients were recruited by the main investigator. The investigators, doctors, nursing staff, and family were blinded to the allocation.

The data were analysed using SPSS version 26 (IBM Corp. Armonk, NY, USA). Independent *t*-tests and Mann–Whitney U tests were used to compare continuous variables between the two groups. Dichotomous variables were compared by the Pearson chi-squared and Fisher exact test.

## 3. Results

**Figure 1:**
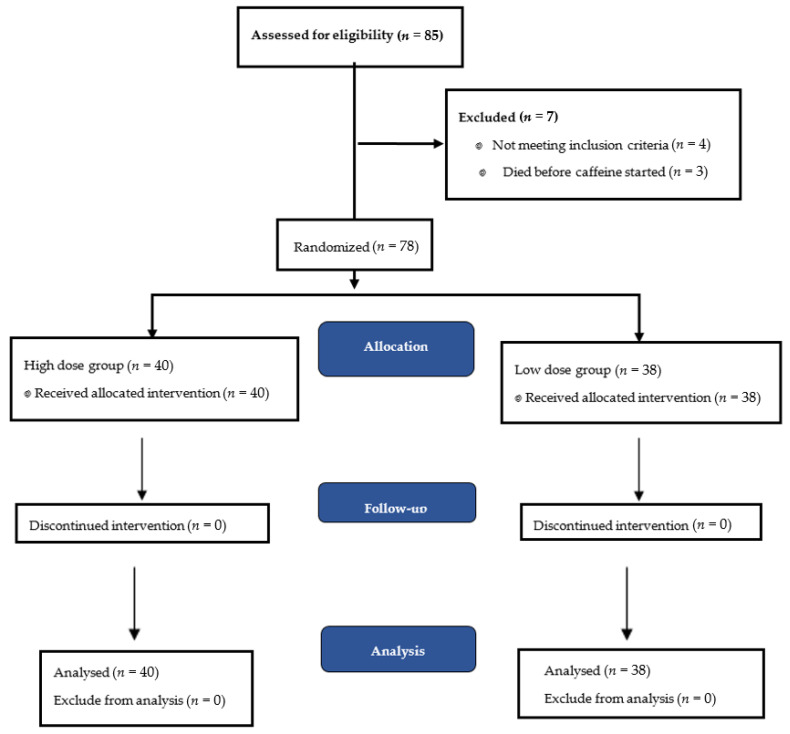
Study enrolment.

Forty infants were assigned to the intervention group and thirty-eight to the control group. All infants received intended treatment until they exited the study. All included infants were analysed for primary outcome. Nine infants (five in the intervention group and four in the control group) received a shortened course of caffeine because of suspected side effects. The flow chart of the enrolment is shown in Figure 1. Among 85 infants assessed for eligibility, four were not included in the study because of the presence of severe congenital abnormalities, and three died before the intervention was started.

The mean birth weight of all participants was 1280 g, and the mean gestational age was 29 weeks. Baseline demographic and clinical characteristics were similar for each group (Table 1).

Table 2 shows the details on the frequency and number of days of apnoea. The majority of infants had no documented apnoea in both groups. There were seven infants who had one to three apnoea episodes in each group. There were six and three infants having four to six apnoea episodes in the intervention and control groups, respectively. Three infants in the intervention group and five infants in the control group had more than six apnoea episodes. There was no difference in frequency and number of days of apnoea between the two groups (*p*-value 0.839 and 0.928, respectively).

Zero Inflated Poisson Regression analysis was performed to identify the influence of intubation on apnoea frequency and the duration of apnoea. The frequency of apnoea episodes among patients on a high dose of caffeine was not significantly different from those on a low dose of caffeine while holding the intubation constant (Coefficient = 0.22; 95% CI: −0.34, 0.79; *p* = 0.443). Similarly, there was no significant difference in the number of days of apnoea between patients on high and those on low doses of caffeine, while holding the intubation constant (Coefficient = 0.04; 95% CI: −0.52, 0.59; *p* = 0.897).

Table 3 shows the secondary outcomes. Among the common side effects of caffeine, tachycardia was seen in 20% and 13% of infants in the intervention and control groups, respectively. Hypertension was seen in 40% and 29%, respectively. The occurrence of these side effects was not statistically significance different between the two groups. No other secondary outcome showed a statistically significant difference between the two groups.

## 4. Discussion

The results of this study suggest that the higher dose of caffeine did not carry an advantage in terms of frequency and number of days of apnoea. Even though no statistically significant difference in side effects was recorded, the absolute number of infants experiencing side effects such as tachycardia and hypertension was higher in the group receiving the higher dose.

The chances of bias in this trial were minimized by a proper randomization, concealment of allocation, blinding of all staff and researchers that could influence the results and reporting of all data collected. The baseline demographic and clinical data collected were similar between the two groups. There may be a large variety of other known and unknown factors influencing the results. It is expected that the randomization resulted in similarity between the two groups such as gravidity and parity of the mothers, previous history of preterm births, and other factors, and that eventual differences for individual factors would balance each other out.

There were no changes made to the methodology or data analysis after the data collection started. A potential source of imprecision may lie in the fact that as shown in several studies [3,15], the incidence of apnoea tends to be under-reported by nurses in their routine recording of clinical data of the babies. If during this study, under-reporting of apnoea would have occurred, it would not have happened to a different degree for each group because all nurses and doctors working in the NICU were blinded to the study allocation. Severe apnoea is unlikely to be under-reported.

This study focused exclusively on short term efficacy and adverse events but did not take into consideration long term outcomes of different caffeine dosage regimens.

The authors feel that the results of this study may be generalizable to many other settings in the world. Even though the study location was in a rural area of Peninsular Malaysia, a high middle-income country, the case mix, available equipment, and staff training and expertise are similar to other middle- and high-income countries. International guidelines and practices were followed for the treatment of preterm babies.

Most previous trials comparing different dosage regimens of caffeine, which were the subject of a review of the literature [16], showed significantly less apnoea with higher dosages. However, when comparing the interventions of this trial with previous trials, the previous trials tended to use caffeine in much lower maintenance doses [17,18,19,20] for the lower dosage regimen. Only one article [21] compared similar dosage regimens as used in the current study, but that study clearly adopted a treatment approach rather than a preventive approach adopted in the current study. Many studies had also a different primary outcome: successful extubation, while the frequency of apnoea was only considered as a secondary outcome [18,19,21,22]. From some of the previous trials, especially the older ones, it is not clear whether the babies received post extubation nasal CPAP or optiflow. A high-quality systematic review of the literature is needed to perform a meta-analysis of all trial results and potentially come up with reliable evidence-based recommendations for practice or for further research. A Cochrane protocol on the dosage of caffeine [23] has been published, but the review is not yet available.

This is the first study comparing high versus low doses of caffeine as a preventive strategy for apnoea in prematurity. The results suggest that the lower dose of caffeine may be as effective as the higher dose in preventing apnoea. As shown in other studies, the side effects of two dosing regimens were not different, even though there was a trend towards a higher number of side effects in the infants given a higher dose.

## 5. Conclusions

In conclusion, this study does not support the usage of high doses of caffeine to prevent apnoea in prematurity. The results of this study can become part of a meta-analysis of all available data, and therefore can contribute to evidence-based practice recommendations for the optimal dosage regimens of caffeine for the prevention of apnoea in prematurity.

## Figures and Tables

**Table 1:** Baseline characteristics of the studied group (*n* = 78).

Characteristics	High-Dose Caffeine	Low Dose Caffeine	*p*-Value
	(*n* = 40)	(*n* = 38)	
Gestational age (weeks)	30 (2.75) ^a^	29.79 (3.32) ^a^	0.244
Birth weight (grams)	1272.75 (415.35) ^b^	1289.47(336.78) ^b^	0.846
Male sex	23 (57.5) ^c^	26 (68.4) ^c^	0.318
Singleton	33 (82.5) ^c^	32 (84.2) ^c^	0.839
Mode of delivery			
Vaginal delivery	18 (45) ^c^	12 (31.6) ^c^	0.846
Caesarean section	22 (55) ^c^	26 (68.4) ^c^	
Small for gestational age			
Yes	6 (15) ^c^	2 (5.3) ^c^	0.264
Maternal chorioamnionitis			
Yes	6 (15) ^c^	6 (15.8) ^c^	0.923
Antenatal steroid therapy			
Yes	28 (70) ^c^	24 (63.2) ^c^	0.522
Intubation			
Yes	33 (82.5) ^c^	37 (97.4) ^c^	0.057
Surfactant			
Yes	32 (80) ^c^	36 (94.7) ^c^	0.088
First age of caffeine treatment (weeks)	30.22 (2.35) ^a^	30.43 (2.39) ^a^	0.572
Duration of caffeine (days)	28.78 (13.22) ^b^	29.89 (14.4) ^b^	0.721
Presence of sepsis			
Yes	10 (25) ^c^	10 (26.3) ^c^	0.894
Number of sepsis	0 (1) ^a^	0 (1) ^a^	0.783
Presence of electrolyte imbalance			
Yes	23 (57.5) ^c^	15 (39.5) ^c^	0.111

^a^ Median (Interquartile range); ^b^ Mean (*SD*); ^c^ Number (%).

**Table 2:** Primary outcome (*n* = 78).

Frequency of Apnoea	High Dose of Caffeine (*n* = 40)	Low Dose of Caffeine (*n* = 38)
0	24	23
1	1	6
2	2	1
3	4	0
4	4	1
5	1	2
6	1	0
7	1	4
8	0	0
9	0	0
10	0	0
11	1	0
12	0	1
13	0	0
14	1	0
Days of apnoea	High dose of caffeine (*n* = 40)	Low dose of caffeine (n) (*n* = 38)
0	24	23
1	3	6
2	4	1
3	6	3
4	1	2
5	0	0
6	1	2
7	1	0
8	0	1

**Table 3:** Secondary outcome (*n* = 78).

Characteristics	High Dose Caffeine	Low Dose Caffeine	*p*-Value
	(*n* = 40)	(*n* = 38)	
Extubation Failure			
Yes	6 (15) ^a^	6 (15.8) ^a^	0.923
Broncho Pulmonary Dysplasia (BPD)			
Yes	10 (25) ^a^	14 (36.8) ^a^	0.257
Duration of Non-Invasive Ventilation (days)	18.50 (32) ^b^	22 (37) ^b^	0.642
Duration of Oxygen Therapy (days)	0 (5) ^b^	0 (7) ^b^	0.752
Systemic steroid for BPD			
Yes	6 (15) ^a^	8(21.1) ^a^	0.486
Time to reach full enteral feeding (day)	12.5 (12) ^b^	13.5 (13) ^b^	0.98
Caffeine withheld for suspected side effect			
Yes	5 (12.5) ^a^	4 (10.5) ^a^	1
Weight gain (g/day)	13.45 (11.92) ^b^	13.38 (10.40) ^b^	0.234
Urine flow rate at day 5 of treatment (ml/kg/hour)	4.60 (1.99) ^b^	4.33 (1.05) ^b^	0.148
Urine Phosphate excretion	4.26 (11.52) ^b^	1.48 (7.64) ^b^	0.557
Urine calcium excretion (mmol/L)	2.21 (3.03) ^b^	1.49 (1.62) ^b^	0.247
Osteopenia of prematurity			
Yes	14 (35) ^a^	16 (42.1) ^a^	0.519
Severe Intraventricular Haemorrhage (IVH) (≥grade 3)			
Yes	0 (0) ^a^	2 (5.3) ^a^	0.234
Retinopathy of Prematurity (ROP)			
Yes	7 (17.9) ^a^	4 (10.5) ^a^	0.352
Necrotising Enterocolitis (NEC)			
Yes	4 (10) ^a^	3 (7.9) ^a^	1
Periventricular Leukomalacia (PVL)			
Yes	2 (5) ^a^	1 (2.6) ^a^	1
Length of Hospital stay (day)	52 (42) ^b^	49.50 (32) ^b^	0.72
Death before hospital discharge	1 (2.5) ^a^	1 (2.6) ^a^	1

^a^ number (percentage); ^b^ median (Interquartile range).

## Data Availability

The data presented in this study are available in the article.
